# Dataset of eye disease-related proteins analyzed using the unfolding mutation screen

**DOI:** 10.1038/sdata.2016.112

**Published:** 2016-12-06

**Authors:** Caitlyn L. McCafferty, Yuri V. Sergeev

**Affiliations:** 1 Ophthalmic Genetics and Visual Function Branch, National Eye Institute, NIH, Bethesda, Maryland 20892, USA

**Keywords:** Retinal diseases, Computational biophysics

## Abstract

A number of genetic diseases are a result of missense mutations in protein structure. These mutations can lead to severe protein destabilization and misfolding. The unfolding mutation screen (UMS) is a computational method that calculates unfolding propensities for every possible missense mutation in a protein structure. The UMS validation demonstrated a good agreement with experimental and phenotypical data. 15 protein structures (a combination of homology models and crystal structures) were analyzed using UMS. The standard and clustered heat maps, and patterned protein structure from the analysis were stored in a UMS library. The library is currently composed of 15 protein structures from 14 inherited eye diseases including retina degenerations, glaucoma, and cataracts, and contains data for 181,110 mutations. The UMS protein library introduces 13 new human models of eye disease related proteins and is the first collection of the consistently calculated unfolding propensities, which could be used as a tool for the express analysis of novel mutations in clinical practice, next generation sequencing, and genotype-to-phenotype relationships in inherited eye disease.

## Background & Summary

For globular proteins, primary protein structure dictates the folds and interactions that occur between amino acids in the structure^1,2^. Genetic mutations lead to protein misfolding and in many cases disease^3^. Protein secondary structure is stabilized by hydrogen bonds from the amide and carboxyl groups of amino acids^4^. The side chains of the amino acids interact in a variety of ways to create the protein tertiary structure (hydrophobic and disulfide interactions). In folding, the protein structure goes through a series of trails and errors to identify the most thermodynamically stable conformation^4^. Therefore, correctly folded proteins have long term stability in biological systems.

The role of missense mutations in inherited disease is not well understood. Disease-causing missense mutations occur when a change at the DNA level causes an amino acid in the protein sequence to be substituted with another, changing the interactions between amino acids and occasionally lead to protein misfolding. Currently, many inherited diseases are caused by missense mutations leading to misfolding of proteins in the cell^3–7^.

UMS is an *in-silico* scan to evaluate the destabilizing effects of multiple point mutations derived from the protein atomic model. It may be used as a tool to analyze the complicated relationship between missense mutations, protein folding, and disease^5^. UMS reads a protein structure file (PDB file) and predicts the unfolding effect for a list of every possible missense mutation that may occur in the protein structure including an identity mutation. For each mutation, UMS calculates an unfolding propensity, derived from the Gibbs free energy equation, to describe whether the mutation will lead to protein misfolding. The output of UMS is a mutational matrix, standard heat map, clustered heat map, and patterned structure. UMS has the ability identify critical residues in the protein structure, which give insight into the most significant residues to protein stability and function. UMS also may explain mutations that can lead to both increased and decreased enzymatic activity, identify trends of residues relating to stability, and predict the severity of missense mutations in disease and their relation to disease phenotype.

Currently there exist a number of protein stability predictors^6–11^. There are also programs that work to predict the functional consequences of missense mutations^12–16^. UMS provides various benefits and advances over current mutant screening techniques. Given that UMS is derived from the atomic structure level and thermodynamics rather than sequence conservation, it has the ability to predict the effect of *de novo* missense mutations^17^. The unfolding propensity is determined using the linear extrapolation model from the normalized sigmoidal unfolding curve obtained experimentally^18^. This data classifies the effects of the missense mutations and uses a universal value so that unfolding propensities from different protein structures may be compared.

The 3 maps and mutation matrix are designed to make this large dataset readable for investigators with different backgrounds such as geneticists, clinicians, biochemists, pharmacologists or protein engineers, and those who may not have any preliminary experience in homology modeling and calculations of protein stability.

Residue depth has been used to describe the protein interior and predict fold types^19–21^. It has been hypothesized that the conservation of ‘deep’ residues is related to folding requirements and function^20^. Relationships exist between highly conserved residues in structural neighbors of the same fold type, and their mean residue depth in the reference structure^21^. There are programs that use residue depth as a parameter to predict protein structural models using fold recognition^19^.

Here, we are reporting the library of 181,110 mutations from 15 proteins from inherited eye disease analyzed with UMS. This analysis includes the preparation of 13 homology models of human proteins. The UMS program has been subjected to intensive validation using the Protherm database and 3 proteins from retinal disease (rhodopsin, complement factor H, and RPE65)^5^. We present 10 new homology models for human proteins related to retinal diseases that have been verified using the internal control. In addition, we provide new maps for each of the 15 proteins for prediction and express analysis of missense mutations. Finally, this study targets a number of new diseases that have not yet been studied using UMS.

## Methods

### Protein preparation

**Figure 1:**
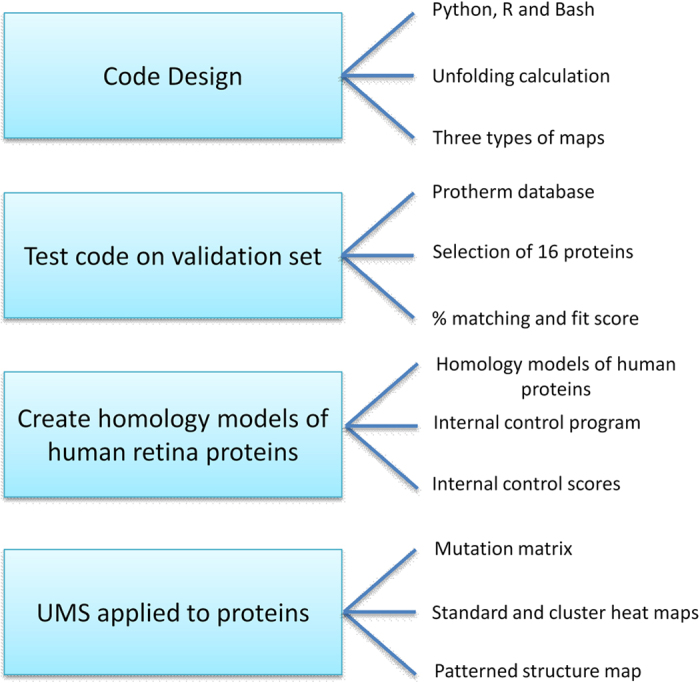
The schematic illustrates the design and testing process used in developing the dataset. The first step was designing the program. The program was written in Python, R and Bash. The purpose of the program was to calculate the unfolding propensity for each missense mutation in a proteins structure. Following, an effective display of the data was pivotal. Next the program was validates using data for 16 proteins obtained from the Protherm database. After the validation human models of proteins related to inherited eye disease were built using homology model. The quality of the structures was tested using the internal control program. Finally UMS was applied to these proteins to produce the mutation matrix, standard and clustered heat maps, and the patterned structure.

A library of 15 different inherited eye disease related proteins was created for analysis. Figure 1 demonstrates the outline of the stages of analysis used. The proteins included in the dataset, pdb names, and their corresponding diseases are shown in Table 1. The human proteins were taken from the RCSB database^22^ or prepared using homology modeling. CYP1B1, IRBP, LRAT, NYX, RDH5, RDH8, RDH12, REP-1, RHO, RPE65, TIMP3, WDR36, and domains 4, 5, 14 and 17 from CFH are homology models. While the remaining crystal structures of CFH domains, CRYAB, and CRYBB1 were obtained from the protein data bank^22^. The pdb files used for the protein analysis are available on the server.

### Internal control

After the homology models were created they were run through the internal control program. The internal control program for the analysis of unfolding propensities is explained in depth in McCafferty & Sergeev^5^. In this work the internal control was adjusted from UMS to calculate the difference in the free energy of the side chain rotamers for the same amino acid. The output of the internal control program was used to either select the best protein models or determine if more refinement of the structures was required. In selecting the best structure, we looked for models with statistically significant data (*P* value <0.05) and then looked for the smallest confidence interval with average close to 0. For those that required further refinement, structures were tested until they fit this requirement.

### UMS program

**Figure 2:**
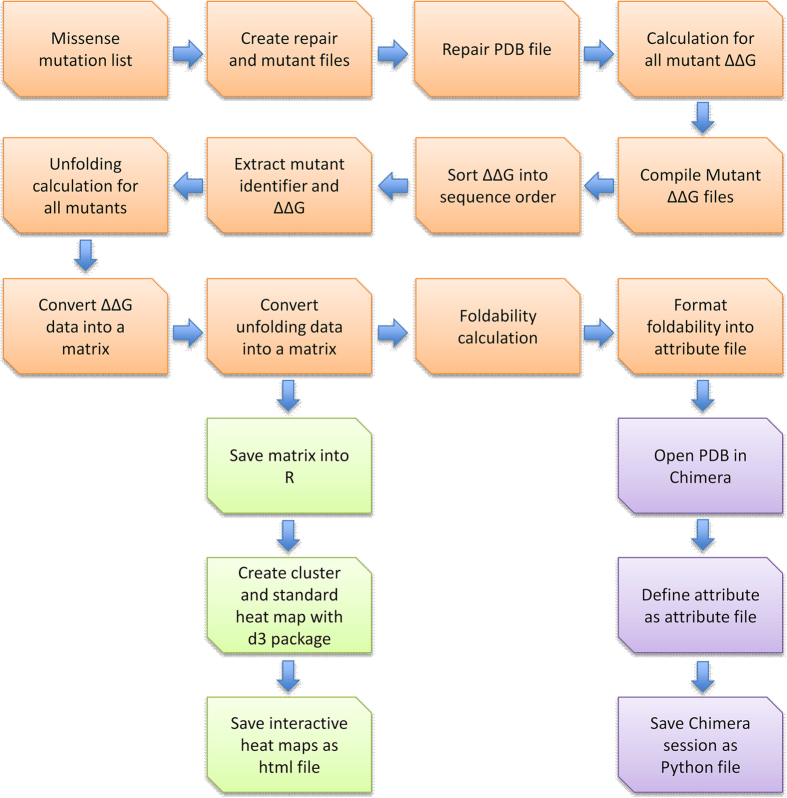
The workflow diagram describes the process of the UMS program. Each function written in the program to carry out the calculations is described. The orange squares represent the operations performed in python, the green squares in R, and the purple in Chimera.

A full description of the UMS program can be found in the methods section of McCafferty & Sergeev^5^. In summary, the program is written in Python, R, and Bash programing languages. The architecture of the program is designed to perform a full mutagenesis analysis as efficiently as possible by implementing a quick and space sensitive procedure. Figure 2 outlines the order of the functions created within the program. The unfolding propensity calculation is derived from the Gibbs free energy equation. The standard and clustered heat maps are produced using the d3heatmap package for R. The maps are interactive and allow specific rows or columns of interest to be selected. Specific region may also be selective for zoomed in view. The clustered maps use an agglomerative, hierarchical clustering method. The groupings are then mapped using a dendrogram. The final map used to convey UMS is the patterned foldability structure. The 3D structures were colored using the foldability value of the residues^5^. These foldability values can then be used to identify critical residues in the protein structure. The critical residues are considered to be essential to proper protein folding.

### Residue depth and informational entropy

In addition, two new descriptors, residue depth and informational entropy, were included as described below. First descriptor, the residue depth of an amino acid in the protein structure, is described as the distance of an atom from the solvent accessible surface^23^. The Biopython package was used to calculate the residue depth for each of the wild type residues in the protein structure^24^. The Biopython package uses the MSMS program for the surface calculation^25^, the residue depth is then presented as the average of the atom depth for each wild type residue in the native protein sequence. The other descriptor, informational entropy, also known as Shannon entropy, quantifies the uncertainty of the source of the information. Therefore, greater informational entropy relates to a greater degree of randomness amongst the mutations for a certain location^26^. For example, if the average unfolding propensity for two locations on the structure were both 0.5, the location where all unfolding propensities were 0.5 would have a lower informational entropy that those that were split between 1.0 and 0. A script was created using Python to calculate the informational entropy of the unfolding propensities. The equation of informational entropy used was $\sum P\left(R=x\right)\times log_{2}\left(\frac{1}{P\left(R=x\right)}\right)$. Both parameters were added to the library to aid the user in studying the relationship between folding and depth within the structure and in analyzing the data provided by UMS, respectively.

### Code availability

The code is available on the *Figshare* (Data citation 1).

## Data Records

**Figure 3:**
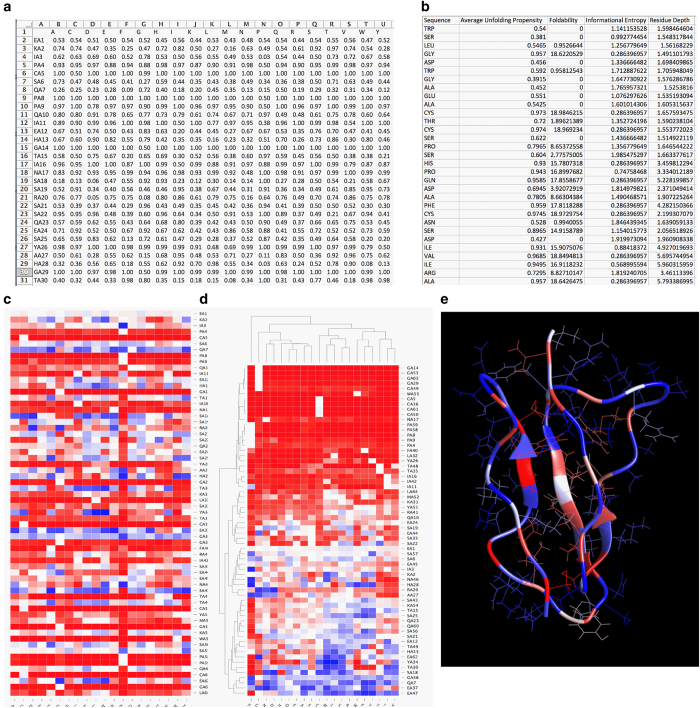
The fragments of data types included for each protein. (**a**) The mutation matrix. The file is in the text format and can be opened in Excel or a text editor. The wild type residues are along the Y-axis and the mutations across the X-axis with the unfolding propensities listed in each box. (**b**) The set of data descriptors for the protein. The first column denotes the wild type sequence, second column the average unfolding propensity at the location, third the foldability, fourth the information entropy, fifth the residue depth. (**c**) The standard heat map. Again, the wild type residues are along the Y-axis and the mutations across the X-axis. The heat map is html file format and is interactive (specific rows/columns/regions may be selected) the red represents the most severe mutations while the blue is the most stabilized. (**d**) The cluster heat map. The file is also html format; here the data is grouped according to an agglomerative hierarchical clustering technique. (**e**) The patterned structure. The structure is a python file that is opened in Chimera. The structure is colored according the foldability values of the residues to highlight the critical residues in the structure.

The UMS library for 15 proteins from inherited eye disease is available on the *Figshare* (Data citation 1). Table 1 presents the PDB file names for each of the proteins included in the study. For each of the proteins analyzed there are five separate files available to describe the data. Figure 3 displays examples of what each of these files looks like. The first is the mutation matrix.

The mutation matrix is available in the protein_matrix.txt format. This can be opened using a standard text editor as well as in Microsoft Excel. The mutation matrix is ideal for an investigator who wants access to the raw data. Since the file can be opened in a number of programs the user has the ability to analyze and manipulate the data however he/she pleases.

The standard and cluster heat maps are available in the protein_standard.html protein_cluster.html format, respectively. The size of each of these maps for the proteins is shown in Table 1. The interactive maps provide facilitated identification of the unfolding propensities even if the protein being studied is large. The html file format allows for the maps to open as a webpage. From here rows and/or columns of interest may be selected. Specific regions may also be highlighted for a zoomed in view. Dragging the mouse over the mutation of interest will reveal the corresponding unfolding propensity. The unfolding propensity ranges from 0–1, where 0 is the most thermodynamically stabile protein, 0.5 is folding-unfolding equilibrium, and 1 is a completely unfolded protein. The standard heat maps are ideal for situations where a specific mutation-unfolding propensity is desired, for example, a clinical setting. Here, each unfolding propensity can be accessed easily. The map can be downloaded and saved for easy reference for a specific patient mutation. The clustered heat map groups residues based on similarity and may be used in studying trends in the protein structure. For example, in structures with disulfide bonds, cysteine residues are clustered together based on the similar destabilization they undergo. This allows us to see the residues that undergo a number of severe mutations or the mutations that have the most harmful effects. A pharmacologist can use this map to identify stabilizing mutations to develop new drugs.

**Figure 4:**
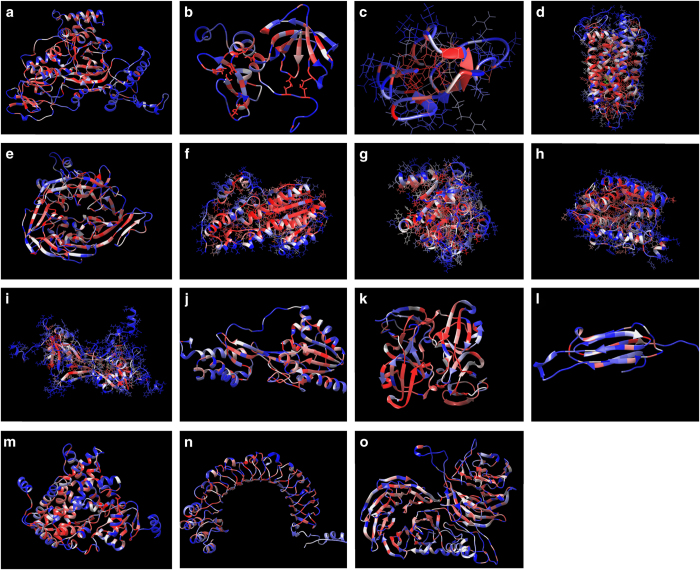
The protein ribbon structures colored by predicted foldability. The structures of proteins related to inherited eye disease. (**a**) REP-1, (**b**) TIMP3, (**c**) sushi domain of CFH, (**d**) rhodopsin, (**e**) RPE65, (**f**) RDH8, (**g**) RDH5, (**h**) RDH12, (**i**) LRAT, (**j**) IRBP, (**k**) CRYBB1, (**l**) CRYAB, (**m**) CRY1B1, (**n**) NYX, (**o**) WDR36.

The patterned structure is available as a python file, protein.py. This file is to be opened using UCSF Chimera (http://www.cgl.ucsf.edu/chimera/). Once opened in Chimera, residues may be identified by placing the mouse over the area of interest. Table 1 also displays the size of the python files to be read by Chimera. This is a 3D map that shows the most critical residues in the protein structure to proper folding. For the particular residue position, foldability could then be used to differentiate between areas that experienced multiple severe mutations, those that experienced a few, and those that had none. Foldability is a more descriptive parameter over simply finding the average in that it can successfully tally all severe mutations that are occurring at a certain location without being influenced by those less severe. Figure 4 shows all of the patterned structures of the inherited eye disease related proteins that were analyzed.

**Figure 5:**
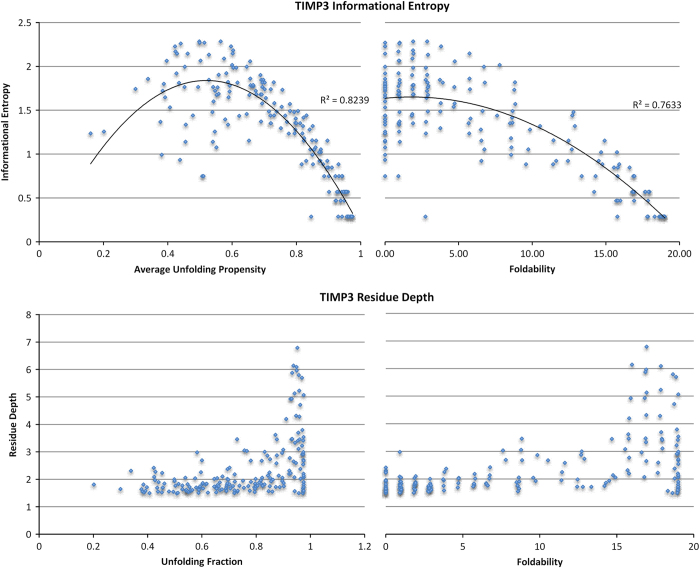
An example of the potential uses for the protein data descriptors. TIMP3 average unfolding propensities and foldability are plotted against the information entropy and residue depth for each wild type location.

Finally, the data descriptor file is again available in the protein_descriptor.txt format. In each column this file contains the native protein sequence, average unfolding propensities, foldability, informational entropy and residues depth (in this order). The descriptor file provides the user some innovation to analyze the data as he/she pleases. Figure 5 shows an example of how one may use this file to analyze the TIMP3 protein. The average unfolding propensity and foldability are plotted against the informational entropy and residue depth.

## Technical Validation

### Validation set criterion (UMS reference)

The validation set for the UMS program was composed of 16 proteins. The proteins were selected from the ProTherm database (http://www.abren.net/protherm/)^27^ based on available experimental thermodynamic data. Proteins with single mutations whose ΔΔ*G* values were determined using fluorescence from denaturants and CD were selected. Specifically, tryptophan fluorescence data for chemical unfolding/refolding in the presence of urea or Gdm-HCl. Proteins with a large number of mutations with thermodynamic data were ideal for the validation. Finally, the proteins needed to have an available PDB file on the Protein Data Bank (http://www.rcsb.org/pdb/)^22^. Based on this criteria the 16 proteins selected for the validation set were: T4 Lysozome (PDB id: 2LZM), Staphylococcal Nuclease (1STN), Protein L (1HZ6), Barnase (1BNI), Ribonuclease T1 Isozyme (1RN1), Gene V Protein (1VBQ), Chymotrypsin Inhibitor 2 (2CI2), Acyl-Coenzyme A (2ABD), Tyrosine-Protein Kinase (1FMK), Acylphosphatase (1APS), Alpha Spectrin (1AJ3), Dihydrofolate Reductase (1RK4), Ribosomal Protein (1RIS), Tryptophan synthase (1WQ5), ARC Repressor (1ARR), and Azurin (5AZU). From the ΔΔ*G* values for each of the experimental mutants from the Protherm database the unfolding propensity was calculated. The percent matching and a Fit-Score were used to evaluate the quality of the output from UMS.

### Homology model validation

**Figure 6:**
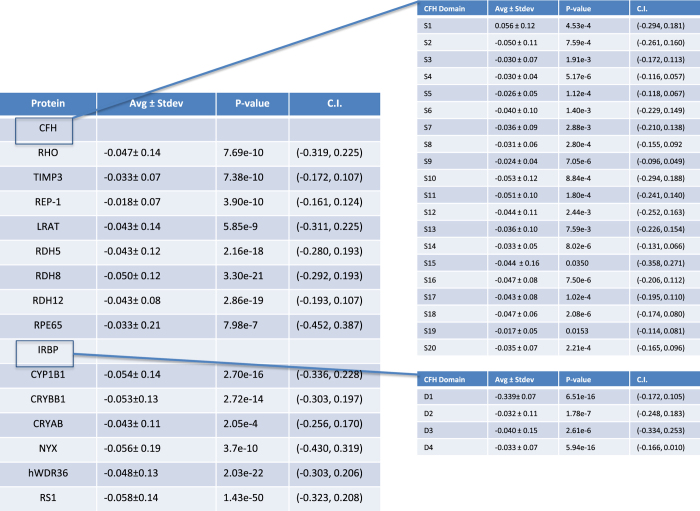
Internal control data. The internal control mutates each residue to itself in order to test the stability of the structure. It is expected that each of these mutations will have a ΔΔG of 0. For each protein the average ΔΔG for all of the mutations is recorded as well as the standard deviation. The *p*-value is also calculated to check to statistical relevance and finally a 95% confidence interval to show the range of the ΔΔG values.

As mentioned in the Methods section an internal control program was designed to validate the homology models used. The results from the internal control are shown in Fig. 6. CFH and IRBP were divided in to their 20 and 4 domains (respectively) in the analysis. The data for each of the proteins fit our criteria for being statistically significant and having small confidence intervals with averages close to 0.

## Usage Notes

All of the protein structures that are included in the dataset are human structures. The homology models, while not crystal structures, have been thoroughly tested for stability and represent models of the human proteins. We aim to eventually create a website of proteins that will constantly be updated and take requests for proteins of interest.

## Additional Information

**How to cite this article**: McCafferty, C. L. and Sergeev Y. V. Dataset of eye disease-related proteins analyzed using the unfolding mutation screen. *Sci. Data* 3:160112 doi: 10.1038/sdata.2016.112 (2016).

**Publisher’s note**: Springer Nature remains neutral with regard to jurisdictional claims in published maps and institutional affiliations.

## Supplementary Material



## Figures and Tables

**Table 1:** UMS output for each protein.

**Protein**	**PDB**	**Mutations Analyzed**	**Disease**	**Standard HM Size**	**Cluster HM Size**	**Patterned Structure Size**
CFH	CFH.pdb	24,320	Age-related Macula Degeneration (AMD)	613 kB	780 kB	1.9 MB
RHO	RHO.pdb	13,920	Retinitis Pigmentosa (RP)	426 kB	477 kB	1.2 MB
TIMP3	TIMP3.pdb	3,940	Sorsby’s fundus distrophy	401 kB	430 kB	334 MB
REP-1	REP-1.pdb	12.54	Choroideremia	487 kB	576 kB	559 kB
LRAT	LRAT.pdb	7,320	Leber’s Congenital Amaurosis (LCA)	424 kB	478 kB	599 kB
RDH5	RDH5.pdb	12,700	Fundus Albipunctatus	464 kB	556 kB	1.1 MB
RDH8	RDH8.pdb	11,880	Myopia, AMD, LCA	453 kB	539 kB	967 kB
RDH12	RDH12.pdb	6,000	LCA, RP, Cone-Rod Dystrophy (CRD)	408 kB	452 kB	499 kB
RPE65	RPE65.pdb	21,320	LCA	544.7 kB	702 kB	2.1 MB
IRBP (domain 1)	IRBP-1.pdb	25,260	arRP	431 kB	477 kB	522 kB
CYP1B1	CYP1B1.pdb	10,160	Glaucoma	470 kB	541 kB	850 kB
CRYBB1	1oki.pdb	7,340	Cataracts	429 kB	481 kB	506 kB
CRYAB	3l1g.pdb	1,900	Cataracts	387 kB	402 kB	322 kB
NYX	NYX.pdb	9,620	Congenital stationary night blindness	463 kB	532 kB	772 kB
WDR36	WDR36.pdb	14,600	Primary Open Angle Glaucoma (POAG)	514 kB	617 kB	1.4 MB
In the table each protein is listed with the PDB file name, the mutations analyzed the inherited disease it is associated with, and the size of each of the map files. The files for other 3 IRBP domains were similar in size (not shown in the Table). In total, 181,110 mutations were analyzed for the 15 proteins.						
